# The Coexistence of Flood and Drought Tolerance: An Opinion on the Development of Climate-Smart Rice

**DOI:** 10.3389/fpls.2022.860802

**Published:** 2022-03-08

**Authors:** A. N. M. Rubaiyath Bin Rahman, Jianhua Zhang

**Affiliations:** ^1^Department of Biology, Hong Kong Baptist University, Kowloon Tong, Hong Kong SAR, China; ^2^State Key Laboratory of Agrobiotechnology, The Chinese University of Hong Kong, Shatin, Hong Kong SAR, China

**Keywords:** drought tolerance, coexistence of flood and drought tolerance, deepwater rice, climate-smart, Bhadoia 303, Rayada rice, Bangladesh, AUS

## Introduction

Despite significant progress in past decades, crop production still depends largely on weather (Iizumi and Ramankutty, 2015). A recent statistical analysis has showed that extreme weather disasters can significantly reduce global crop production. Over the past five decades, drought and extreme heat reduced national cereal production by about 10% (Lesk et al., 2016). Climate models predict that the frequency, intensity, and impacts of some extreme weather events are likely to increase in the future. Simply put, climate change brings a cascade of risks including the risk and uncertainty in crop production. Therefore, the development and release of climate-resilient crop varieties are vital to cope with the challenges of the impending population coupled with the advance of climate change. Water stress (either shortage or excess) can be the most damaging of all abiotic stresses in rice cultivation if occurred at specific stages. For example, droughts at flowering stage, more specifically, from 3 weeks before flowering to 1 week after anthesis, can significantly reduce rice production due to spikelet sterility (Ekanayake et al., 1989). Similarly, most rice varieties are highly susceptible to flooding stress; rice plants die if submerged for more than a week (Bailey-Serres et al., 2010).

Drought and flooding stresses are commonly viewed as distinctly different abiotic stresses as both stresses are studied, analyzed and even reviewed separately (Bin Rahman and Zhang, 2016). However, both drought and flooding stresses can occur sequentially during a single crop cycle. Rice cultivation in rainfed lowland areas (i.e., one third of the total rice production area in the world) have become increasingly threatened by the effects of climate change. Therefore, we need climate-smart rice varieties tolerant to both drought and flooding stresses without substantial yield penalty if no stress occurs.

Plant genetic resources are the basic raw materials for any crop improvement program. Fortunately, rice is one of the unique crops that has cultivars/accessions tolerant to all forms of flooding stresses. Genetic bases of different types of flood tolerance mediated by *SUBMERGENCE 1* (*SUB1A-1*), *SNORKEL1* (*SK1*)*, SNORKEL2* (*SK2*), *TREHALOSE 6-PHOSPHATE PHOSPHATASE 7* (*TPP7*), deepwater rice–specific variant of *SEMIDWARF1* (*SD1-DW*) etc. have been revealed in the past two decades (Xu et al., 2006; Hattori et al., 2009; Kretzschmar et al., 2015; Kuroha et al., 2018). The tolerant features of submergence and anaerobic germination have already been introgressed into modern rice varieties and released to farmers in a number of Asian countries (Bailey-Serres et al., 2010). Unlike monogenic traits, introgression of polygenic traits like drought tolerance in modern varieties is relatively difficult due to the linkage drag, which often brings undesired traits (like the reduction in quality, yield, etc.) from the donor parent together with the favorable genes/QTLs.

Rice has special ecotypes, generally called deepwater rice, cultivated in flood-plain deeper than 50 cm for 1 month or longer during the growing season (Cattling, 1992). Deepwater rice utilize *SK1*/*SK2* (Hattori et al., 2009) and deepwater rice–specific variant of *SEMIDWARF1* (*SD1-DW*) (Kuroha et al., 2018) for rapid internode elongation to keep top leaves above water. Previous studies identified two deepwater rice types, Bhadoia/Ashwina and Rayada as distinct varietal groups, alike Indica and Japonica (Glaszmann, 1987). Among the deepwater rice, Rayada rice has a year-long life cycle, hence Rayada rice experiences all annual stress conditions (cold, drought, flooding stress) in a single crop cycle (Bin Rahman and Zhang, 2013). Numerous deepwater rice varieties (e.g., Shail Kota, Manikdigha, etc.) were identified with pronounced drought tolerance capacity over some well-accepted drought-tolerant upland varieties (Cattling, 1992). Similarly, a number of deepwater rice varieties such as Sarsari, Bhabani, Hijaldigha etc. were identified with excellent recovery capacity from drought stress (Datta and O'Toole, 1977). However, in-depth exploration of deepwater rice varieties for drought-tolerant gene mining has always been neglected (Bin Rahman and Zhang, 2013).

## The Coexistence of Flood and Drought Tolerance

In 2016, we proposed a counterintuitive hypothesis on the coexistence of flood and drought tolerance in rice (Bin Rahman and Zhang, 2016). According to our hypothesis, flood and drought tolerance may coexist in aus subpopulation and the molecular mechanisms of drought and flood tolerance may be regulated by cross-talking signaling pathways. We developed the hypothesis after analyzing the distribution and population types of drought and flood-tolerant rice accessions together with the molecular mechanisms of their tolerances (Bin Rahman and Zhang, 2016). After its publication, this paper attracted global attention and has now been cited from all major rice growing countries. A number of notable scientists picked up the findings. For instance, Davies and Ribaut (2017) remarked the findings as good news for the development of climate-resilient rice varieties.

Remarkably, recent results of a collaborative study conducted by International Rice Research Institute (IRRI) and New York University, published in the Plant Cell (Groen et al., 2021), supported both parts of our hypothesis. While studying the patterns of rice adaptation to drought-prone agro-ecosystems using evolutionary systems biology approach, Groen and his colleagues identified Bhadoia 303, a deepwater rice accession from the aus subpopulation, as the most drought-responsive accession with the highest number of differentially expressed genes. The highest number of uniquely expressed genes was also observed in Bhadoia 303 among six cultivars/accessions. The uniquely expressed genes in Bhadoia 303 comprised almost one third (32.6%) of the differentially expressed genes (Groen et al., 2021). In our hypothesis, we argued for ethylene-mediated shared pathways for both flood and drought tolerance. A similar pattern of differential activity of APETALA2/ETHYLENE RESPONSIVE FACTOR (AP2/ERF) -family transcription factors was observed in drought-stressed shoot samples in the recent study. Remarkably, Bhadoia 303 showed the least yield reduction among other varieties, compared to its well-watered condition in the field experiments (Groen et al., 2021).

Bhadoias are early type deepwater rice, named after the Bengali calendar month, Bhadra (August–September) when they usually flower (Bin Rahman and Zhang, 2013). Bhadoias are only grown in lowlands in Bangladesh and adjoining states of India where the maximum water depth does not exceed 1.50 meters. Unlike typical deepwater rice, Bhadoia varieties are photoperiod insensitive and usually flower months earlier than typical deepwater rice varieties (Bin Rahman and Zhang, 2013). Although earlier studies identified Bhadoia as a distinct varietal type (group III) (Glaszmann, 1987), recent population studies categorize Bhadoia as *circum-aus* (Wang et al., 2018).

## Prospects: A Concerted Research Effort to Develop Climate-Smart Rice Varieties

After the recent confirmation of our hypothesis, we believe it is high time to identify the genomic speciality of drought tolerance in deepwater rice and mine the key genes/QTLs from Bhadoia 303 and other deepwater rice varieties*/*accessions. A concerted research effort (summarized below) is necessary to develop climate-smart rice varieties for rained-fed agro-ecosystems.

### Need More Systems Biology Studies With Drought Exposure at Reproductive Stage

In our opinion, more systems biology studies integrating morphological, molecular and physiological responses to water deficits are necessary for better understanding of underlying mechanisms of drought tolerance developed among rice sub-populations. This approach may close the translation gap between laboratory findings and field performance (Groen and Purugganan, 2016). However, instead of the vegetative stage, water stress should be imposed at the reproductive stage. We know drought responses between vegetative and reproductive stages are different (Kamoshita et al., 2008) where vegetative stage drought tolerance does not correlate with yield performance under water stress. Direct selection for yield performance in both drought and well-watered conditions has been increasingly accepted for the selection/identification of drought tolerant breeding lines/varieties (Kumar et al., 2008; Venuprasad et al., 2008; Torres et al., 2013). Therefore, water deficits should be imposed at the reproductive stage in future systems biology studies.

### Need Research Collaboration Between Rice Research Institutes and Universities

As the occurrence of natural droughts in rainfed lowland and upland rice fields is unpredictable, a controlled drought environment in field condition (such as rain-out shelters) is necessary in screening for drought tolerance. The severity and timing of droughts can be easily controlled in rain-out shelters. However, most universities do not possess sufficient resources to handle large field studies, which require special facilities like rain-out shelters, a large number of trained-technicians for simultaneous uniform sampling, etc. Oppositely, rice research institutes usually possess such facilities, expertise, etc., but are typically less interested in basic research. Therefore, we think more research collaboration between rice research institutes and universities may speed up the development of climate-smart rice varieties. The identification of the genetic basis of submergence tolerance (Xu et al., 2006) and the development of submergence tolerant rice varieties exemplify successful research collaboration between rice research institutes and universities.

### Need Deep Exploration of Deepwater Rice Varieties of Bangladesh

Bangladesh is the hub of the deepwater rice diversity (Bin Rahman and Zhang, 2013). We believe it is high time to explore more deepwater rice varieties, particularly from Bhadoia and Rayada types, for their speciality in stress tolerance. The distribution/cultivation of both types of deepwater rice are confined to Bangladesh (and some adjacent Indian states) (Bin Rahman and Zhang, 2013) and previous studies identified them as two distinct varietal groups (group III and IV) (Glaszmann, 1987). Using detailed sequencing analyses, Kuroha et al. (2018) revealed that deepwater rice–specific variant of *SEMIDWARF1* (*SD1-DW*) is predominantly available in deepwater rice varieties of Bangladesh whereas deepwater rice in other countries only harbor *SK1*/*2*. Moreover, we have earlier showed that abiotic stress tolerance accessions are predominately originated from Bangladesh (Bin Rahman and Zhang, 2018). Similarly, we know numerous other Bhadoia accessions beside Bhadoia 303, such as Bhadoia 648 (IRGC 6502), Bhadoia 688 (IRGC 6492), Bhadoia 689 (IRGC 6536), etc. were identified as highly drought tolerant accessions in previous drought screening. Unlike traditional deepwater rice varieties, Bhadoias are photoperiod insensitive, therefore, they can be easily incorporated in the studies of direct yield selection, alike the recent one (Groen et al., 2021). However, the inclusion of highly photoperiod sensitive varieties like Rayada requires special attention for sowing date, daylength of the experiment sites, etc. for synchronized flowering with other varieties.

### Need More Focus on Root Traits

Drought tolerance is often labeled as being a very “complex trait” in molecular and genomics studies (Blum, 2011). However, experienced agronomists, physiologists or breeders' perspective on drought tolerance is much simpler; the deeper the roots, the better the tolerance (Gowda et al., 2011; Henry et al., 2011; Uga et al., 2013). However, the extraction of roots from drought stressed field is much more difficult than collecting above-ground leaf/shoot samples, requiring pickaxes and hammers to crack open rock-hard soil. Using both drought-stressed root and shoot samples, Groen et al. (2021) not only showed the association of root traits with rice plant fitness under drought but also revealed some unique patterns in the drought-stressed roots. Transcription factor binding sites enriched in promoters of co-expression modules between drought-stressed shoot and root samples was significantly different. Unlike AP2/ERFs in shoots, bHLH (and bZIP) transcription factor binding sites are enriched in promoters of root co-expression modules (Groen et al., 2021). It is well evident that droughts have a much more pronounced effect on the root transcriptome. Therefore, we believe future drought research should focus on root traits, particularly root growth at depth (i.e., beneath a 20-cm depth), which often explain a large proportion of the drought response in rice (Henry et al., 2011; Gowda et al., 2012; Uga et al., 2013).

Finally, we conclude the opinion on a positive note. The development and release of rice varieties tolerant to both drought and flooding stresses have already started. Recently, five rice varieties tolerant to both drought and submergence have been released to farmers in India (Sukkha Dhan 6, CR Dhan 801, CR Dhan 802) and Nepal (Sukha Dhan 6, Bahuguni dhan-1, and Bahuguni dhan-2) (Pradhan et al., 2019; Sandhu et al., 2019). Similarly, improved deepwater rice varieties are also released in Bangladesh (BRRI 91 in 2019), India (Rajdeep CN 1039-9 in 2017), Thailand (RD45 in 2010). Hopefully, rice farmers will be able to cultivate climate-resilient multi-stress tolerant rice varieties in near future. The cultivation of climate resilient crop varieties along with the adoption of modern agricultural practices like silicon fertilization (Thorne et al., 2020; Thakral et al., 2021) etc. are essential for sustainable crop production with the advance of climate change.

## Author Contributions

AB and JZ: conceptualization and writing—review and editing. AB: writing—original draft. JZ: supervision and correspondence. Both authors contributed to the article and approved the submitted version.

## Funding

This work was supported by the Hong Kong Research Grant council with a GRF grant (GRF 12103219) to JZ.

## Conflict of Interest

The authors declare that the research was conducted in the absence of any commercial or financial relationships that could be construed as a potential conflict of interest.

## Publisher's Note

All claims expressed in this article are solely those of the authors and do not necessarily represent those of their affiliated organizations, or those of the publisher, the editors and the reviewers. Any product that may be evaluated in this article, or claim that may be made by its manufacturer, is not guaranteed or endorsed by the publisher.
